# Supplementary material for: Asymmetric Synthesis of Quaternary Hydantoins via a Palladium-Catalyzed Aza-Heck Cyclization
Source: J Am Chem Soc. 2025 Nov 14;147(49):44692–8. doi: 10.1021/jacs.5c16022 (PMC12703750; doi:10.1021/jacs.5c16022)
Supplement: Supplementary file 2 [file ja5c16022_si_002.zip › All NMR FID Files/23/23_ProtonNMR/1/pdata/1/email_EM05-231-1-1H_1_1.pdf]

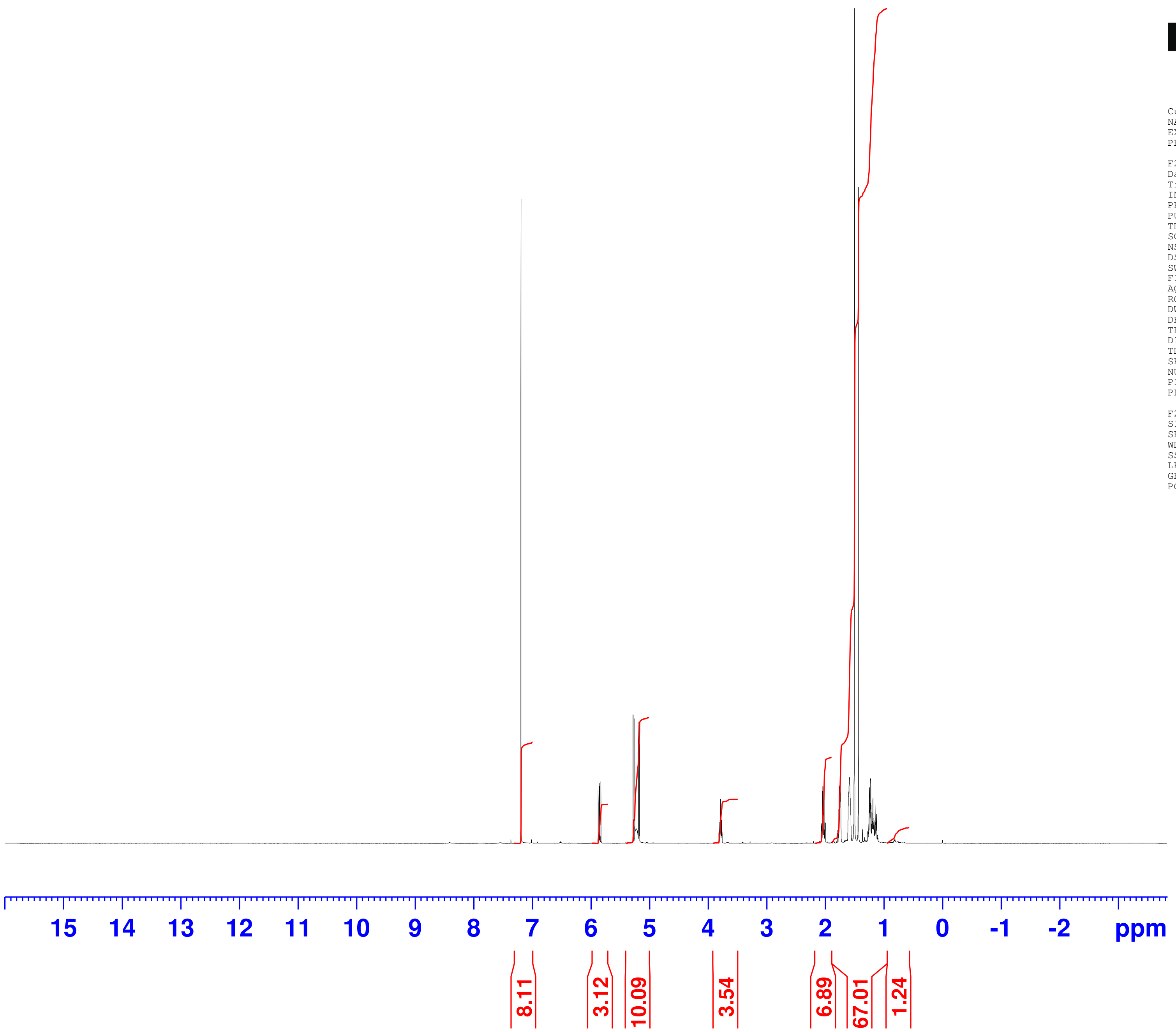


```
Current Data Parameters
NAME        EM05-231-1-1H
EXPNO                   1
PROCNO                  1

F2 - Acquisition Parameters
Date_            20240924
Time                10.37 h
INSTRUM  Avance Neo 600 at University of Delaware
PROBHD   Z185170_0001 (
PULPROG                zg
TD                  65536
SOLVENT             CDCl3
NS                     16
DS                      2
SWH             11904.762 Hz
FIDRES           0.363304 Hz
AQ              2.7525120 sec
RG                   11.3
DW                 42.000 usec
DE                  10.61 usec
TE                  298.1 K
D1            10.00000000 sec
TD0                     1
SFO1          600.1337058 MHz
NUC1                   1H
P1                   8.07 usec
PLW1           6.90000010 W

F2 - Processing parameters
SI                  65536
SF            600.1300557 MHz
WDW                    EM
SSB                     0
LB                   0.30 Hz
GB                      0
PC                   1.00
```
